# Mental health considerations for children & adolescents in COVID-19 Pandemic

**DOI:** 10.12669/pjms.36.COVID19-S4.2759

**Published:** 2020-05

**Authors:** Nazish Imran, Muhammad Zeshan, Zainab Pervaiz

**Affiliations:** 1 Dr. Nazish Imran, MBBS; FRCPsych (London); MRCPsych (London); MHPE Associate Professor, Department of Child & Family Psychiatry, King Edward Medical University/Mayo Hospital, Lahore, Pakistan; 2 Dr. Muhammad Zeshan, MD, Child Psychiatry Fellowship (Harvard) Assistant Professor, Psychiatry, Attending Child Psychiatrist Rutgers New Jersey Medical School, Newark, NJ, USA; 3 Dr. Zainab Pervaiz, MBBS Post Graduate Resident, Academic Department of Psychiatry & Behavioural Sciences, King Edward Medical University Lahore, Pakistan

**Keywords:** Adolescents, Children, Corona virus, COVID-19, Mental health, Pandemic

## Abstract

Children are not indifferent to the significant psychological impact of the COVID-19 Pandemic. They experience fears, uncertainties, substantial changes to their routines, physical and social isolation alongside high level of parental stress. Understanding their emotions and responses is essential to properly address their needs during this pandemic. In this article, we highlight children’s vulnerability, provide an overview of common symptoms of distress in different age groups, and summarize the interventions and resources available to promote child mental health and wellbeing during these challenging times. We advocate that prioritizing mental health including child & adolescent mental health is an essential component of any universal, community led response to COVID-19 Pandemic.

## INTRODUCTION

The outbreak of the Novel Coronavirus (COVID-19) in December 2019 started in China and it was declared a pandemic in March 2020, by World Health Organization. It has affected more than 200 countries around the world with total number of COVID-19 patients being more than two million and number of deaths reported to be 177,338 as of 22^nd^ April, 2020.^1^ Pakistan has also been affected by the pandemic with more than 14,788 patients and 322 deaths till April 28, 2020.^1^ Although the number of children affected by the disease is small, and most of the affected children show only mild symptoms,^2^ the disease and the containment measures are likely to negatively impact the mental health & well-being of children. Even though children all over the world are going to be affected, those with disabilities, living in slums, isolation centers, and conflicts zones are going to be at a greater risk. This article provides an insight into the mental health challenges faced by children in the wake of recent COVID-19 pandemic.

### Significance of child and adolescent mental health in relation to pandemics

Public health emergencies like pandemics, take a toll on physical as well as mental health. Children are particularly vulnerable because of their limited understanding of the event. They are unable to escape the harms of the situation physically and mentally as they have limited coping strategies. They may not be able to communicate their feelings like the adults. Closure of schools and separation from friends can cause stress and anxiety in children. Exposure to mass media coverage of crisis event and unverified information circulating on social media may aggravate the mental distress.^3^,^4^

Response of a child to a crisis situation depends upon his prior exposure to emergency situations, his physical and mental health, socio-economic circumstances of the family, and cultural background.^3^,^4^ Different studies have shown that crisis events negatively impact the psychological wellbeing of children. Anxiety, depression, disturbances in sleep and appetite as well as impairment in social interactions are the most common presentations. A recent study conducted in China screened children and adolescents for behavioral and emotional distress due to the Covid-19 pandemic. Clinginess, distraction, irritability, and fear that family members can contract the deadly disease were the most common behavioral problems identified.^5^

### Impact of current disease containment measures on child mental health

Pakistan, like many other countries, has asked its citizens to practice social distancing in order to combat the spread of Covid-19. Educational institutions have been closed, exams have been postponed, shopping malls, restaurants, and all areas of public gathering are under a strict lockdown. The rapid rise in the number of infected cases and deaths, disruption of daily routines, home confinement, fear of infection, social distancing from peers and friends, and lack of access to educational resources have created a feeling of uncertainty and anxiety among the children and the adolescents.^4^ Disease containment measures though necessary, can adversely affect child & families’ well-being due to various reasons.

### Increase Screen time

In these testing times of social isolation, children and teens are becoming exposed to the excessive media coverage of the pandemic. On one hand, electronic and social media is providing continuous updates on nationwide situation, and advising people to adopt social distancing but on the other hand, it is also creating sensationalism and spreading misinformation. The screen time of children and adolescents has increased manifold as they are confined to their homes. Research has shown that after 9/11, excessive television exposure led to increased incidences of Post-Traumatic Stress Disorder (PTSD) and other mental health disorders.^6^ There are fears that similar disorders can develop in children due to excessive usage of electronic and social media. Furthermore, excessive social media usage makes children vulnerable to online predators, cyberbullying and potentially harmful content.

### Parental Stress

Many families are isolated at home, under great stress, and unable to receive in-person support. Confinement, social isolation, and inability to use familiar coping mechanisms like taking personal space, visiting with family/ friends, or going to mall or movie theatre, dining out or going for a long drive on car or motorcycle with spouse or friend may exacerbate the impact of these stressors.

Stable, supportive, and nurturing caregiver relationship offer young children fostering trust, positive social-emotional development, and the capacity to form a secure and strong relationship in the future.^7^ A disaster such as the COVID-19 pandemic may cause parents and caregivers to become fearful and worried about their own concerns about health and the inability to meet demanding economic needs especially in developing countries like Pakistan. Extensive research shows that fear can be contagious and children are extremely sensitive to the emotional state of the adults around them, who are their essential source of security and emotional well-being. The pandemic has disrupted the financial and economic activities around the world creating a sense of uncertainty among the masses. A survey conducted in USA indicates high level of psychological distress among people suffering from financial losses. Around 40% of the people with children less than 12 years of age fall into high distress group and are having difficulties in dealing with their work responsibilities and child care.^8^ More than 50% of the parents have reported that financial troubles due to social isolation are affecting their parenting skills.^9^ As the Holy month of Ramadan has already started and celebration of Eid-ul-Fitr is approaching soon, inability to fulfill children’s wishes and demands are likely to further compound financial problems and parental stress.

### Risk of Child Abuse, Neglect, & Exploitation

Unfortunately, social distancing measures can result in social isolation in an abusive home, with abuse likely to be exacerbated during this time of financial/social instability, fear of infection, boredom, and frustration. Study from previous financial recessions, natural disasters, and outbreaks like the Ebola outbreak in West Africa from 2014-2016, revealed increased rates of child abuse, neglect, and exploitation. There have been recent reports from Jianli County in Hubei province, China, that police report of domestic violence was increased to more than triple during the lockdown in February 2020, from 47 last years to 162 this year. Moreover, some reports of a surge in suspected child abuse cases in the state of Texas in United States have also been noted.^10^-^12^ In another survey, parents have reported increased incidents of yelling, shouting and even slapping their children .^9^ Chris Newlin, the executive director of the National Children’s Advocacy Center, USA, stated that more than 80% of children, who are sexually abused, know the perpetrator. Children who have had histories of previous trauma, have had any mental and or physical health problems, have parents who are divorced/separated or incarcerated or parents who have mental/chronic physical health problems are more vulnerable to abuse. Staying home may make these children more prone to undergo further mistreatment, neglect, child maltreatment, gender-based violence, and exploitation.

### Common symptoms of Psychological Distress in children during Pandemics Younger children

Young children sense their parents’ stress and may display their worries in ways that caregivers may interpret as misbehavior, oppositional/defiant behavior, and temper tantrum. Parents may notice that their toddlers and preschoolers are more fussing and winning, are struggling to focus or engage in play, and are becoming more aggressive. Some children may start showing typical regressive behaviors like asking for bottle, thumb sucking, toileting accidents, not wanting to dress or feed themselves, becoming clingier and demanding, wanted to be carried, as well as problem in sleeping. The sleep pattern changes may include problem going to sleep, multiple awakening in the middle of the night, frequent nightmares, not being able to take naps during the day, as well as demanding more attention at nap and bedtime. These stress reactions in children may cause parental self-doubt and feeling inadequate, difficulty understanding and empathizing, increasing sense of sadness, depression and lack of control, sleep deprivation, caregivers’ withdrawal and shutting down, and as well as may trigger parental trauma or stress response.

### Older children/Teenagers

Older children and adolescent may feel disappointed for missing birthday parties, school plays, dance competitions, hanging out with their friends, sport activities like playing cricket in playground with other team members, as well as not being able to visit their grandparents, aunts, friends, and cousins. As articulated by psychologist Erik Erikson, Identity vs Role Confusion is the fifth of eight stages of psychosocial development that takes place between the ages of twelve and nineteen.^13^ Important event during this stage is social relationship. Teenagers and college students have amplified energy, novelty, motivation, curiosity, and enthusiasm that make them hard to isolate at home. The hormonal changes that come with puberty collude with adolescent social dynamics to make them highly attuned to social status, peer group, and relationships. Teens may feel frustrated, nervous, disconnected, nostalgic, and bored because of social distancing during this pandemic.

### Children with special needs

Children with special needs are particularly vulnerable to the negative psychological impacts of disasters.^14^ School closures can have significant impact on the lives of those with special needs. Children with autism spectrum disorder and neurocognitive disability can become frustrated due to disruptions in their daily routines.^15^ Their regular therapy sessions may get interrupted and they are more likely to show problematic behaviors such as irritability, aggression and social withdrawal.^16^

### Children with Pre-existing Mental Illnesses

Children suffering from depression and anxiety disorders may feel overwhelmed with the news of death and disease all around them. The obsessions and compulsions of a child with OCD may get worse during the times of stress. In short disease mitigation measures and the disease, itself can be severely traumatic for the children with preexisting psychiatric disorders.

### Children in Quarantine

Children quarantined under the suspicion of having covid-19 or diagnosed with the disease are likely to develop mental health disorders such as anxiety, acute stress, and adjustment disorders.^17^ Separation from parents, stigmatization, fear of an unknown disease, and social isolation can all have a negative psychological impact on children. Studies show that negative psychological impact from quarantine can be detected even after months and years.^18^ A study shows four times increase in mean post-traumatic stress scores in quarantined children compared to non- quarantined children.^18^ Another study indicated that 30% of the children quarantined fulfilled the criteria of PTSD.^19^

### Interventions for promoting child mental health during COVID-19 General Interventions

Although all the world health agencies and governments are working tirelessly to contain the deadly virus, much more needs to be done to combat the mental and psychological impact of the disease. Nevertheless, several world health agencies including WHO, UNICEF, AACAP, IACAPAP, and many others have issued guidelines and factsheets to help parents safeguard the mental health of their children in these testing times.

These guidelines are based on the basic principles of reassuring the children, educating them about the situation in age appropriate ways, and maintaining daily routines. Other guidelines include educating children on maintaining safe distances and practicing personal hygiene, acknowledging their distress and answering all their questions with honesty. Parents should avoid unnecessary separation from children and if separated from parents, children should have alternate care givers available, and they should be able to contact parents regularly. Exposure of children to panic provoking news on media should be avoided and positive use of social media should be encouraged e.g. to form support groups etc. Furthermore, screen time of children should also be monitored.^20^

### Specific Interventions

**a)Younger children**.
  - If the child is demanding more attention at nap time and/or bedtime, try to spend more quality time with him/her/them during the day, by possibly taking 10-15 minutes’ breaks where parents focus entirely on the child. Enjoy blowing bubbles, listening to music or singing/dancing at home.
  - Setting up a routine for family.
  - Turn off the news channels when young children are around. They will hear things or see images or videos that are potentially scary.
  - Refrain from talking about the COVID related situation with other adults or older siblings around them.
  - Younger kids may need a bit more hugs and cuddles than older kids. If parents are concerned about transmitting illness, then sitting close, or perhaps sleeping in the same room might be worth trying.
  - Encourage younger children to adequately and frequently wash hands or wipe surfaces of any playful game.
  - Encourage video chat. Video chat helps young children—even babies—remember and build relationships with family members and other caregivers.
  - Try to talk about what is happening on the screen while watching programs or playing video games with your children. Although children may engage with screens more than usual during this pandemic, but make sure they have mix of activities across the day, including story/book time, free play, art activities, some active play like hide & seek, running around, jumping on a trampoline, copying/imitating baby facial or body gestures, building train tracks, or riding a tricycle.
**b)Older Children:**
  - Set times for a few regular activities each day such as home tutoring, telephone calls with a friend, or cooking together, family meals; do these activities at the same time each day
  - Make sure they spend some time outdoors every day, or do some exercise daily. If one can’t go outside, try to spend at least two hours by a window, looking into the daylight, and focusing on being calm.
  - Social interactions are important, even during social distancing; videoconferencing, telephone, or real-time text-messaging may be worth to consider, possibly at the same time each day.
  - Avoid frequent day time naps, especially later in the day; if they must nap, restrict the nap to 30 minutes—napping can make it hard to go to sleep at night.
  - Avoid bright (especially blue) light like computer screens, smartphones in the evening. Blue spectrum light suppresses the hormone that helps us sleep.
  - Advise adolescents to stick to a consistent sleep and wake time that fits their natural rhythms.
  - If child is sad about not having a birthday party, or missing an important social event, validate his/her feeling of sadness and frustration, acknowledges their losses, listen empathically to their thoughts, feeling and emotions, and collaboratively explore some possible solutions. Arranging a surprise birthday party with family and friends on Zoom might not sound too crazy!
**c)Children with Special Needs:**
  - For children with special needs (ASD/ID), excessive disruptions in daily routines should be avoided by encouraging them to have daily schedule of activities that can be carried out in home environment. Communication should be maintained with their therapists and schools.^15^
**d)Children in quarantine:**
  - Children in quarantine should be able to contact their parents frequently. They should be guided to maintain daily routine and should have access to disease information. They should be able to contact mental health professionals if need arises.
**e)Some other Important consideration for families:**
  - Create some stable consistent routines that nurture the whole family. Mental Health Professionals often emphasize on how bedtime and mealtime routines help children feel safe, organized, and secure. But routines help parents and adults feel that way too!
  - Disease containment measures like social isolation have enabled families to spend more time together. Many Parents have reported increased sense of closeness and intimacy with their children.^11^ Make use of this quality time by reading a book or making drawings with them, engaging them in house hold chores like cleaning and cooking, and occupy them with indoor scavenger hunts, board and card games to reduce the stress and boredom due to home confinement.
  - During stressful times, children need a safe, reassuring, and secure relationship with their caregivers whom they can express their feelings and questions.
  - Use simple, developmentally appropriate language (especially for young children) to explain rationale for social distancing, not being able to visit friend’s/ family members/grandparents, needs to wear facial masks etc. Some possible simple statements could be any of the following:
    1. We cannot play with other kids lately so that we can stay healthy.
    2. We wipe things down (including door knobs, toys, furniture etc.) to keep them clean
    3. Sometime people wear masks when they are not feeling well. When they start feeling well, they will stop wearing masks.
    4. Everyone can get sick anytime. If you or grandpa/grandma get sick, mama and papa will take good care of all of you until we all get better
  - Society as a whole, need to play an active role to ensure that vulnerable population like children, adolescents and elderly are supported during this challenging time by responsible media coverage and following of social distancing guidelines by Government.
  - Lastly the parents and care givers should take care of themselves as well. If they are confident and free of stress, they may be able to guide, educate and protect their children more effectively and efficiently.

## CONCLUSION

Ignoring the immediate and long-term psychological effects of COVID-19 Pandemic would be disastrous, especially for children and young people, who account for almost 50% of population in Pakistan. Interventions need to focus on nurturing resilience in children and adolescents by better communication to address their fears and concerns, encouraging routines and physical activities, and taking measures to alleviate loneliness. Parents need to look after their own mental health, coping strategies, and model positive psychological attitude in order to support children and adolescents to get through this difficult time.

### Author`s Contribution

**NI** conceived the review, did literature search, participated in write-up, coordination, and she also responsible and accountable for the accuracy or integrity of the study.

**MZ** participated in literature review, helped in manuscript writing.

**ZP** helped in literature review, write-up & critical revision.

All authors read and approved the final manuscript.
